# Effect of intensity-modulated radiotherapy versus two-dimensional conventional radiotherapy alone in nasopharyngeal carcinoma

**DOI:** 10.18632/oncotarget.8573

**Published:** 2016-04-04

**Authors:** Pu-Yun OuYang, Dingbo Shi, Rui Sun, Yu-Jia Zhu, Yao Xiao, Lu-Ning Zhang, Xu-Hui Zhang, Ze-Ying Chen, Xiao-Wen Lan, Jie Tang, Yuan-Hong Gao, Jun Ma, Wuguo Deng, Fang-Yun Xie

**Affiliations:** ^1^ Department of Radiation Oncology, Sun Yat-sen University Cancer Center, State Key Laboratory of Oncology in South China, Collaborative Innovation Center for Cancer Medicine, Guangzhou, Guangdong, China; ^2^ Department of Experimental Research, Sun Yat-sen University Cancer Center, State Key Laboratory of Oncology in South China, Collaborative Innovation Center for Cancer Medicine, Guangzhou, Guangdong, China; ^3^ Department of Nasopharyngeal Carcinoma, Sun Yat-sen University Cancer Center, State Key Laboratory of Oncology in South China, Collaborative Innovation Center for Cancer Medicine, Guangzhou, Guangdong, China

**Keywords:** intensity-modulated radiotherapy, nasopharyngeal carcinoma, propensity score matching, two-dimensional conventional radiotherapy

## Abstract

**Background:**

Albeit intensity-modulated radiotherapy (IMRT) is currently the recommended radiation technique in treating nasopharyngeal carcinoma, the effect of IMRT versus two-dimensional conventional radiotherapy (2DCRT) alone is still contradictory.

**Results:**

In the original unmatched cohort of 1198 patients, IMRT obtained comparable 5-year overall survival (OS) (91.3% vs 87.1%, *P* = 0.120), locoregional relapse-free survival (LRFS) (92.3% vs 90.4%, *P* = 0.221) and distant metastasis-free survival (DMFS) (92.9% vs 92.1%, *P* = 0.901) to 2DCRT. In the propensity-matched cohort of 604 patients, no significant survival differences were observed between the two arms (5-year OS 90.9% vs 90.5%, *P* = 0.655; LRFS 92.5% vs 92.4%, *P* = 0.866; DMFS 92.5% vs 92.9%, *P* = 0.384). In multivariate analysis, IMRT did not significantly lower the risk of death, locoregional relapse or distant metastasis, irrespective of tumor stage.

**Methods:**

Overall, 1198 patients who underwent IMRT (316 patients) or 2DCRT (882 patients) without any chemotherapy was retrospectively analyzed. Patients in both arms were matched at equal ratio using propensity-score matching method. OS, LRFS and DMFS were assessed with Kaplan-Meier method, log-rank test and Cox regression.

**Conclusions:**

In this propensity-matched study, IMRT showed no survival advantage over 2DCRT alone in nasopharyngeal carcinoma.

## INTRODUCTION

Nasopharyngeal carcinoma (NPC) is a distinct type of head and neck cancer, relatively rare in Europe and the United States [1] but highly endemic in Southern China [2] and Hong Kong [3]. Radiotherapy is the cornerstone of initial treatment. Over the past few years, a shift toward the adoption of novel radiation techniques has been witnessed. Intensity-modulated radiotherapy (IMRT) rapidly replaced two-dimensional conventional radiotherapy (2DCRT), and now it represents the most commonly used radiation option for NPC. It is expected to lower the rates of treatment-related toxicity and simultaneously improve survival, irrespective of a concomitant substantial increase in expenditures.

A retrospective study [4] indicated advantage of IMRT over 2DCRT only in local control of stage T1. Inversely, a prospective study [5] observed higher local control in stage T4, better regional control in stage N2 and improved overall survival (OS) in stage III, especially in stage N2. In a recent retrospective study [6] with long term follow up, IMRT was reported to significantly enhance local control in stage T1-4 and regional control in stage N1, prolong locoregional relapse-free survival (LRFS) and progression free survival in stage I-IV and improve OS in stage II-III. These contradictory results did not provide accurate information regarding the effect of IMRT versus 2DCRT. Additionally, none of these studies could totally exclude the interference of chemotherapy. Since it is unethical and impracticable to prospectively compare IMRT and 2DCRT in locoregionally advanced patients without any type of chemotherapy in a randomized controlled trial, we sought to retrospectively assess the survival differences across these radiation techniques in a large cohort of patients who underwent radiotherapy alone. Particularly, patients in the IMRT and 2DCRT arms were well matched with balanced characteristics using propensity score matching method to mimic randomized trials [7].

## RESULTS

### Patients

A total of 1198 patients were included. Respectively, 316 and 882 patients were treated with IMRT and 2DCRT alone. In comparison with patients who underwent 2DCRT, those received IMRT had significant younger age, lower titer of immunoglobulin A against early antigen (EA-IgA), early N-stage and early clinical stage (*P* ≤ 0.006). Following propensity score matching, 302 pairs of patients treated with IMRT or 2DCRT alone were identified with highly balanced characteristics (standardized difference ≤ 0.077). (Table 1)

**Table 1:** Baseline characteristics of nasopharyngeal carcinoma patients treated with intensity-modulated radiotherapy or two-dimensional conventional radiotherapy

	The original unmatched cohort						The propensity-matched cohort					
	IMRT (*N*=316)		2DCRT (*N*=882)		*P*	Standardized difference	IMRT (*N*=302)		2DCRT (*N*=302)		*P*	Standardized difference
	No.	%	No.	%			No.	%	No.	%		
**Age**					**0.005**	**0.182**					**0.702**	**0.031**
Mean	47.41		49.67				47.61		47.98			
SD	12.65		12.14				12.71		11.05			
Median	46.00		49.00				46.00		47.00			
**Sex**					**0.217**	**0.082**					**0.771**	**0.024**
Male	244	77.2	650	73.7			232	76.8	235	77.8		
Female	72	22.8	232	26.3			70	23.2	67	22.2		
Histology*					**0.123**	**0.105**					**0.513**	**0.053**
II	18	5.7	74	8.4			18	6.0	22	7.3		
III	298	94.3	808	91.6			284	94.0	280	92.7		
**VCA-IgA**^†^					**0.229**						**0.951**	
<80	82	25.9	201	22.8		**0.074**	80	26.5	77	25.5		**0.023**
80-320	127	40.2	336	38.1		**0.043**	118	39.1	118	39.1		**0.000**
≥320	107	33.9	345	39.1		**0.109**	104	34.4	107	35.4		**0.021**
**EA-IgA**^†^					**0.006**						**0.715**	
<10	138	43.7	302	34.2		**0.194**	126	41.7	123	40.7		**0.020**
10-40	103	32.6	305	34.6		**0.042**	102	33.8	111	36.8		**0.062**
≥40	75	23.7	275	31.2		**0.167**	74	24.5	68	22.5		**0.047**
**T-stage**					**0.076**						**0.635**	
T1	133	42.1	317	35.9		**0.126**	127	42.1	122	40.4		**0.034**
T2	98	31.0	279	31.6		**0.013**	92	30.5	99	32.8		**0.050**
T3	66	20.9	197	22.3		**0.035**	64	21.2	56	18.5		**0.066**
T4	19	6.0	89	10.1		**0.150**	19	6.3	25	8.3		**0.077**
**N-stage**					**<0.001**						**0.700**	
N0	169	53.5	369	41.8		**0.235**	162	53.6	155	51.3		**0.046**
N1	120	38.0	443	50.2		**0.249**	115	38.1	126	41.7		**0.074**
N2	22	7.0	68	7.7		**0.029**	21	7.0	19	6.3		**0.027**
N3	5	1.6	2	0.2		**0.144**	4	1.3	2	0.7		**0.067**
**Clinical stage**					**0.004**						**0.671**	
I	96	30.4	181	20.5		**0.228**	91	30.1	82	27.2		**0.066**
II	120	38.0	377	42.7		**0.097**	114	37.7	125	41.1		**0.075**
III	76	24.1	233	26.4		**0.055**	74	24.5	68	22.5		**0.047**
IV	24	7.6	91	10.3		**0.095**	23	7.6	27	8.9		**0.048**

Abbreviations: IMRT = intensity-modulated radiotherapy, 2DCRT = two-dimensional conventional radiotherapy, SD = standard deviation, VCA = viral capsid antigen, EA = early antigen, IgA = immunoglobulin A

* Based on the criteria of WHO histological type (1991): II - Differentiated non-keratinising carcinoma, III - Undifferentiated non-keratinising carcinoma

† In accordance with the criteria adopted in previous studies

### Survival outcomes

**Figure 1:**
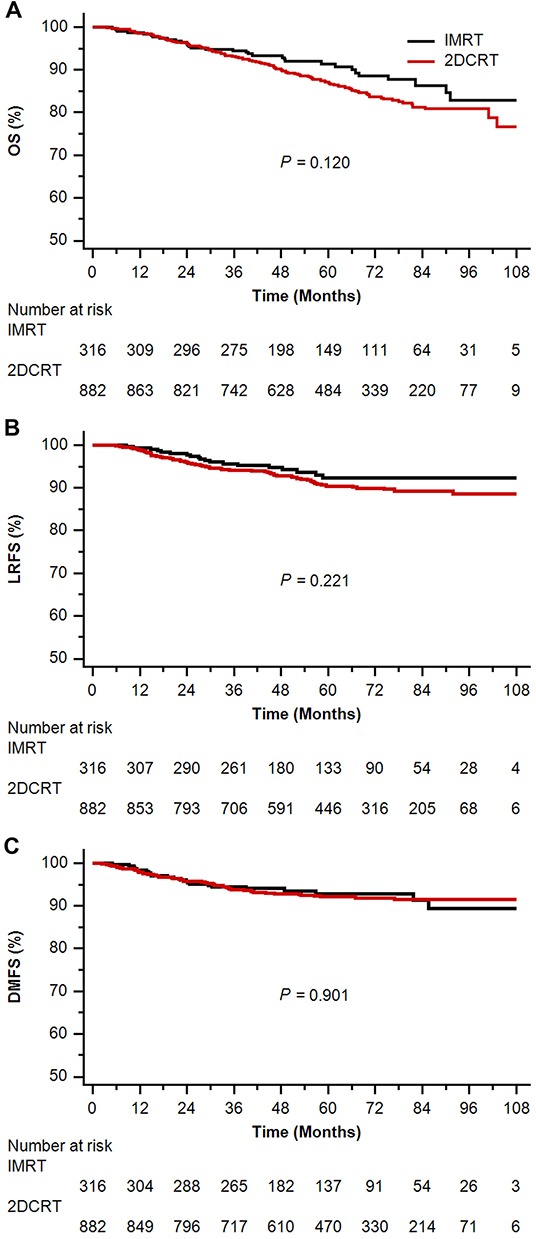
Kaplan-Meier survival curves of intensity-modulated radiotherapy (IMRT) arm versus two-dimensional conventional radiotherapy (2DCRT) arm in the original unmatched cohort **A.** overall survival (OS); **B.** locoregional relapse-free survival (LRFS); **C.** distant metastasis-free survival (DMFS).

In the original unmatched cohort, median follow-up time was 55.80 months (4.37-115.70 months) in the IMRT arm and 63.05 months (3.60-117.90 months) in the 2DCRT arm, respectively. Compared with 2DCRT alone, IMRT did not significantly improve OS, LRFS or distant metastasis-free survival (DMFS) (5-year OS 91.3% vs 87.1%, *P* = 0.120; LRFS 92.3% vs 90.4%, *P* = 0.221; DMFS 92.9% vs 92.1%, *P* = 0.901; Figure 1). And the insignificant differences between the two arms were sustained when adjusted for age (continuous), sex, histology, immunoglobulin A against viral capsid antigen (VCA-IgA), immunoglobulin A against early antigen (EA-IgA), T-stage and N-stage (all *P* ≥ 0.240) (Table 2). Considering the great impact of tumor stage on survival, we did second analysis by tumor stage. Among patients with stage I and II, IMRT showed similar 5-year OS (98.2% vs 94.4%, *P* = 0.120), LRFS (93.6% vs 92.6%, *P* = 0.369) and DMFS (98.6% vs 95.7%, *P* = 0.268) to 2DCRT. And IMRT also failed to prolong the 5-year OS (76.2% vs 74.2%, *P* = 0.839), LRFS (89.0% vs 86.5%, *P* = 0.488) and DMFS (79.0% vs 85.5%, *P* = 0.247) of patients with stage III and IV. Irrespective of T-stage, N-stage or clinical stage, IMRT showed no survival advantage over 2DCRT in multivariate analysis (adjusted *P* ≥ 0.146). (Table 3)

**Table 2:** Summary of significant prognostic factors in multivariate analysis

	The original unmatched cohort		The propensity-matched cohort	
	Hazard ratio (95% CI)	*P*^*†*^	Hazard ratio (95% CI)	*P*^*‡*^
**Overall survival**				
IMRT versus 2DCRT	1.26 (0.86-1.87)	0.240	1.31 (0.78-2.20)	0.313
Age (continuous)	1.06 (1.05-1.07)	<0.001	1.06 (1.04-1.09)	<0.001
T-stage	1.79 (1.51-2.11)	<0.001	1.72 (1.28-2.33)	<0.001
N-stage	2.09 (1.67-2.61)	<0.001	2.02 (1.42-2.88)	<0.001
**Locoregional relapse-free survival**				
IMRT versus 2DCRT	1.25 (0.76-2.07)	0.381	1.20 (0.64-2.23)	0.576
T-stage	1.36 (1.10-1.68)	0.004	1.23 (0.90-1.67)	0.194
N-stage	1.67 (1.24-2.25)	0.001	1.89 (1.29-2.77)	0.001
**Distant metastasis-free survival**				
IMRT versus 2DCRT	0.89 (0.55-1.44)	0.629	0.98 (0.53-1.81)	0.939
Age (continuous)	1.02 (1.01-1.04)	0.005	1.03 (1.00-1.05)	0.028
T-stage	1.84 (1.47-2.30)	<0.001	2.06 (1.54-2.77)	<0.001
N-stage	2.43 (1.80-3.29)	<0.001	2.26 (1.54-3.32)	<0.001

Abbreviations: CI = confidence interval, 2DCRT = two-dimensional conventional radiotherapy, IMRT = intensity-modulated radiotherapy

† Adjusted for age (continuous), sex, histology, immunoglobulin A against viral capsid antigen (<80/80-320/≥320), immunoglobulin A against early antigen (<10/10-40/≥40), T-stage and N-stage by forward selection.

‡ Adjusted for the same covariates by forward selection with a robust variance estimator to account for the clustering within matched pair.

**Table 3:** IMRT versus 2DCRT in subgroup analysis by tumor stage in multivariate analysis in the original unmatched cohort*

Subgroup	Overall survival		Locoregional relapse-free survival		Distant metastasis-free survival	
	Hazard ratio (95% CI)	*P*	Hazard ratio (95% CI)	*P*	Hazard ratio (95% CI)	*P*
**T-stage**						
T1-2	1.24 (0.66-2.35)	0.503	1.08 (0.58-2.02)	0.802	1.95 (0.75-5.05)	0.170
T3-4	1.15 (0.70-1.90)	0.574	1.78 (0.74-4.30)	0.200	0.65 (0.36-1.16)	0.146
**N-stage**						
N0-1	1.28 (0.80-2.03)	0.302	1.26 (0.73-2.19)	0.402	0.84 (0.48-1.48)	0.543
N2-3	1.03 (0.47-2.26)	0.945	1.12 (0.31-4.07)	0.869	1.06 (0.39-2.87)	0.908
**Clinical stage**						
I+II	1.43 (0.69-2.97)	0.336	1.22 (0.63-2.38)	0.559	1.49 (0.56-3.94)	0.421
III+IV	1.13 (0.71-1.80)	0.600	1.29 (0.60-2.78)	0.521	0.72 (0.41-1.26)	0.250

Abbreviations: IMRT = intensity-modulated radiotherapy, 2DCRT = two-dimensional conventional radiotherapy, CI = confidence interval

* Adjusted for age (continuous), sex, histology, immunoglobulin A against viral capsid antigen (<80/80-320/≥320), immunoglobulin A against early antigen (<10/10-40/≥40), T-stage and N-stage by forward selection.

**Figure 2:**
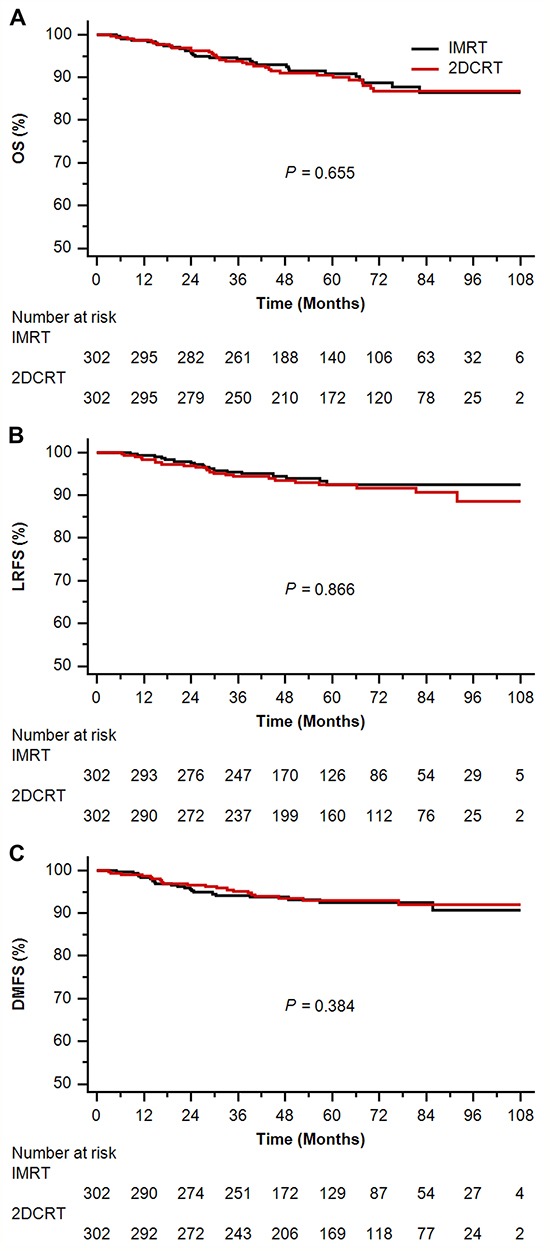
Kaplan-Meier survival curves of intensity-modulated radiotherapy (IMRT) arm versus two-dimensional conventional radiotherapy (2DCRT) arm in the propensity-matched cohort **A.** overall survival (OS); **B.** locoregional relapse-free survival (LRFS); **C.** distant metastasis-free survival (DMFS).

In the propensity-matched cohort, median follow-up time was 55.12 months (4.37-115.70 months) in the IMRT arm and 64.43 months (3.60-109.93 months) in the 2DCRT arm, respectively. In univariate analysis, IMRT resulted in parallel survival to 2DCRT (5-year OS 90.9% vs 90.5%, *P* = 0.655; LRFS 92.5% vs 92.4%, *P* = 0.866; DMFS 92.5% vs 92.9%, *P* = 0.384; Figure 2). Adjusting for the known prognostic factors, IMRT showed similar efficiency to 2DCRT in management of death, locoregional relapse and distant metastasis (adjusted *P* ≥ 0.313) (Table 2). In subgroups of patients with stage I and II, IMRT achieved comparable 5-year OS (98.1% vs 95.9%, *P* = 0.414), LRFS (94.1% vs 94.0%, *P* = 0.819) and DMFS (98.5% vs 95.0%, *P* = 1.000) to 2DCRT. Similarly, IMRT was analogous to 2DCRT in 5-year OS (75.3% vs 78.5%, *P* = 0.127), LRFS (88.5% vs 88.8%, *P* = 0.739) and DMFS (78.1% vs 88.3%, *P* = 0.225) in subgroups of patients with stage III and IV. In multivariate analysis, IMRT had no benefit in OS, LRFS or DMFS versus 2DCRT, regardless of T-stage, N-stage and clinical stage (adjusted *P* ≥ 0.102) (Table 4).

**Table 4:** IMRT versus 2DCRT in subgroup analysis by tumor stage in multivariate analysis in the propensity-matched cohort*

Subgroup	Overall survival		Locoregional relapse-free survival		Distant metastasis-free survival	
	Hazard ratio (95% CI)	*P*	Hazard ratio (95% CI)	*P*	Hazard ratio (95% CI)	*P*
**T-stage**						
T1-2	1.27 (0.51-3.17)	0.608	1.12 (0.51-2.44)	0.780	2.51 (0.79-7.93)	0.118
T3-4	1.27 (0.65-2.50)	0.481	1.49 (0.49-4.57)	0.486	0.62 (0.28-1.38)	0.243
**N-stage**						
N0-1	1.59 (0.89-2.87)	0.119	1.28 (0.64-2.56)	0.481	1.21 (0.63-2.32)	0.572
N2-3	0.43 (0.16-1.18)	0.102	0.90 (0.23-3.55)	0.878	0.36 (0.06-2.37)	0.290
**Clinical stage**						
I+II	1.65 (0.61-4.47)	0.325	1.44 (0.60-3.46)	0.411	2.34 (0.71-7.69)	0.161
III+IV	1.17 (0.62-2.21)	0.633	1.07 (0.42-2.72)	0.890	0.63 (0.28-1.38)	0.245

Abbreviations: IMRT = intensity-modulated radiotherapy, 2DCRT = two-dimensional conventional radiotherapy, CI = confidence interval

* Adjusted for age (continuous), sex, histology, immunoglobulin A against viral capsid antigen (<80/80-320/≥320), immunoglobulin A against early antigen (<10/10-40/≥40), T-stage and N-stage by forward selection with a robust variance estimator to account for the clustering within matched pair.

## DISCUSSION

The current study released the first report regarding the effect of IMRT versus 2DCRT for NPC patients without chemotherapy. IMRT showed no advantage over 2DCRT in locoregional control, distant metastasis or OS, irrespective of the disease of T-stage, N-stage or clinical stage.

This was not the first null report. By head to head comparison, Fang et al [8] and Moretto et al [9] both found that IMRT was comparable to 2DCRT in locoregional control, DMFS and OS, despite in smaller cohorts of patients. In a phase III randomized trial [10] aiming at the efficiency of adjuvant chemotherapy in locoregionally advanced NPC, radiation technique (IMRT vs 2DCRT/3DCRT) was also not associated with any type of survival. Conversely, it was slightly different from other reports [4–6]. The absence of chemotherapy in our study maybe primarily caused the differences. As chemotherapy, regardless of neoadjuvant, concurrent or adjuvant chemotherapy, can reduce tumor volume, enhance radiosensitivity or lower the risk of distant metastasis, locoregional relapse and treatment failure [11], it is hard to exactly evaluate the role of radiotherapy in a cohort with the assistance of chemotherapy. Even though patients were randomly assigned to IMRT or 2DCRT in the prospective study by Peng et al [5], various sequences and regimens of cytotoxic drugs were used in both arms and the magnitude of survival differences caused by heterogeneous chemotherapy was unknown as a result. Secondly, propensity score matching method highly balanced the heterogeneity in certain known prognostic factors, such as sex [12] and tumor stage [13], which helped to eliminate the observed interference in the retrospective comparison of IMRT and 2DCRT [4, 6]. Additionally, our findings, if analyzed in terms of tumor stage, were actually supported by previous studies. For instance, IMRT showed similar nodal relapse-free survival, DMFS and disease free survival (DFS) to 2DCRT, irrespective of N-stage and clinical stage, and IMRT was comparable to 2DCRT in local control among patients with T2-4; the only benefit obtained by IMRT was a higher local control rate in patients with stage T1, as demonstrated in study by Lai et al [4]. Likewise, Peng et al [5] observed no advantage of IMRT versus 2DCRT in OS, except for those patients with stage III.

Notably, about 70% of patients in this study were diagnosed with NPC of early stage, which matched the relative higher survival outcomes in comparison with prior reports [4–6, 8, 9] on the whole. When stratified by tumor stage, however, the outcomes of early stage patients treated with IMRT and 2DCRT were highly similar to the results of the studies by Su et al [14] and Zhang et al [6], respectively. As for patients with stage III and IV, results of IMRT alone were reasonably lower than those of IMRT plus chemotherapy[15], while the outcomes of 2DCRT were superior to the published studies, such as the study by Chen et al [16]. This was most likely to be determined by the limited tumor extension and low tumor burden that the majority of patients in this radiotherapy alone cohort had.

Chau et al [17] once reported that the D(95) of the primary tumor increased from 57.1 Gy (2DCRT) to 67 Gy (IMRT) and from 45 Gy (2DCRT) to 63.6 Gy (IMRT), respectively, in the case of NPC staged T3–4, and that the mean maximum dose delivered to critical structures was reduced from 61.8 Gy (2DCRT) to 52.8 Gy (IMRT) and from 56 Gy (2DCRT) to 43.6Gy (IMRT), respectively. Hence, IMRT appears to facilitate dose escalation, spare the surrounding critical normal tissues and theoretically obtain better local control as a result. However, since the supplement of intracavitary intubation [18] or stereotactic radiotherapy boost [19, 20] to 2DCRT actually helped to achieve excellent local control as well, it is not irrational to find similar LRFS between IMRT and 2DCRT in our study. Certainly, the effect of 2DCRT herein was in fact the combined one of 2DCRT and intracavitary intubation or radiotherapy boost. As reported by Li et al [21], IMRT had the same distant metastatic timing and distribution as 2DCRT, and consequently it had limited contribution to distant control in NPC. In combination, IMRT was not unexpected to achieve equivalent survival outcomes to 2DCRT, as showed in the present study.

The major strength of this study lies in the comparison of IMRT and 2DCRT alone in a large scale cohort of NPC patients using propensity score matching and multivariate analysis. The presented data was derived from a single institution in endemic area with expertise in diagnosing and treating this disease, this provided the utility in treatment. Since data on DNA copy number of the Epstein-Barr virus was missing in most of cases, VCA-IgA and EA-IgA were taken as the surrogate. The independent effect of intracavitary intubation or radiotherapy boost was not specially evaluated herein, because it had been previously confirmed on one hand, and on the other, it was once delivered as the supplementary treatment to conventional 2DCRT and should be evaluated in the combined form. It is a limitation that some patients might be delayed in detecting lung metastasis and consequently have falsely high DMFS rate, owing to the low sensitivity rate of chest radiography compared with chest computed tomography (CT). But the intrinsic differences in DMFS might scarcely change, as the chance of delay was equal to patients in both arms. Additionally, most of included patients were diagnosed with early stage NPC, so the effect of IMRT versus 2DCRT alone in locoregionally advanced disease needed more sufficient investigation in the future.

In conclusion, this propensity-matched study indicated no significant differences in survival between IMRT and 2DCRT for NPC patients without any chemotherapy.

## MATERIALS AND METHODS

### Patients

Between Mar 2004 and Jan 2011, 1198 biopsy-proven, non-metastatic and treatment-naïve NPC patients who were at the age of 20 or above were included. All patients had complete pretreatment evaluation including patient history, physical examination, hematology and biochemistry profiles, fiberoptic nasopharyngoscopy with biopsy, magnetic resonance imaging (MRI) of the nasopharynx and neck, chest radiography or CT, abdominal sonography or CT, and Technetium-99m-methylene diphosphonate (Tc-99-MDP) whole-body bone scan or CT/MRI of bones. Patients were restaged in accordance with the 2010 International Union against Cancer/American Joint Committee on Cancer (UICC/AJCC) staging system for NPC.

### Treatment

All patients were treated by definitive IMRT or 2DCRT alone. The cumulative radiation doses were 66 Gy or greater to the primary tumor, 60 Gy or greater to the involved cervical lymph nodes and 50 Gy or greater to potential sites of local infiltration and bilateral cervical lymphatics in 30-33 fractions. Further details of the radiation technique have been described previously [15].

### Follow-up

Patients were examined every 3–6 months during the first 3 years, and every 6–12 months thereafter until death. During this period, patients were assessed by history and physical examination and a series of conventional examination equipment (e.g., fiberoptic nasopharyngoscopy, MRI of the nasopharynx and neck, chest radiography or CT, abdominal sonography or CT, and Tc-99-MDP whole-body bones scan or CT/MRI of bones, etc.) at each follow-up visit, to detect the possible relapse or distant metastasis. Confirmed locoregional relapse, distant metastasis and/or consistent disease were treated with irradiation, surgery and/or chemotherapy. Patients without recent examination tests in the medical records were followed up by telephone call.

### Statistical analysis

Patients treated with IMRT were selected to match those treated with 2DCRT using propensity score matching method. This method creates similar case (IMRT) and control (2DCRT) arms, and reduces possible biases to a minimum in a retrospective analysis [22]. Propensity scores were computed by logistic regression for each patient based on the following covariates, age, sex, histology (WHO II, differentiated non-keratinising carcinoma; WHO III, undifferentiated non-keratinising carcinoma [23]), VCA-IgA, EA-IgA, T-stage, N-stage and clinical stage. Patients were then matched without replacement at the ratio of 1:1 on those scores, rather than the individual covariates. Covariates balance between the two sets were examined by *t* test (continuous variable), χ^2^ test (categorical variable) and standardized difference [24] for the original unmatched and propensity-matched cohorts.

OS (time from treatment to death from any cause), LRFS (time from treatment to the first locoregional relapse) and DMFS (time from treatment to the first distant metastasis) were estimated with the Kaplan–Meier method [25] and compared with log-rank test. Adjusted hazard ratios with 95% confidence intervals were calculated using Cox proportional hazards model [26]. In the propensity-matched cohort, survival curves were compared using stratified log-rank test by matched pairs, and hazard ratios were estimated using Cox proportional hazards model with a robust variance estimator to account for the clustering within matched pairs [27].

All statistical analyses were performed using IBM SPSS Statistics version 23.0 and Stata version 13.0. Two-sided *P* values < 0.05 and standardized difference > 0.10 [28] were considered to be significantly different.
